# Identifying the landscape and contribution of advanced nurse practitioners in supporting healthcare provision in Ireland in the 21st century: An integrative review

**DOI:** 10.1016/j.ijnsa.2025.100304

**Published:** 2025-01-30

**Authors:** Owen Doody, Margaret Graham, Orla Hegarty, Anne Marie Sloane, Pauline Walsh, Mary Synnott, Mary Russell, Trudy Dunworth, Jill Sheridan, Louise Murphy

**Affiliations:** a Health Research Institute, University of Limerick, Limerick, Ireland; b School of Nursing & Midwifery, University of Limerick, Limerick, Ireland; c HSE Mid West Mental Health Services, St. Joseph's Hospital, Mulgrave Street, Limerick, Ireland

**Keywords:** Advanced nurse practitioner, Advanced practitioner, Ireland, Contribution, Role, Professional practice, Integrative review, Healthcare provision

## Abstract

**Background:**

In Ireland the role of advanced nurse practitioner has developed significantly since 2001. This evolution is rooted in the growing recognition of the need for highly skilled nursing professionals to address complex healthcare demands and improve patient outcomes.

**Objective:**

To scope the landscape and identify the effect of advanced nurse practitioners on healthcare provision in Ireland.

**Design:**

A systematic search of eight academic databases (CINAHL, Embase, PsycINFO, Scopus, Medline and Academic Search Complete, Cochrane, Web of Science) relevant to nursing and health care were performed.

**Setting(s):**

Nursing care environment.

**Participants:**

Advanced nurse practitioners delivering care.

**Methods:**

A pre-defined systematic search of eight academic databases was conducted, and two reviewers screened each study against the inclusion criteria. Additional hand-searching of the reference lists (backward chaining) and citations (forward chaining) of papers that met the inclusion criteria was conducted. The methodological details of each paper were extracted and assessed for quality and rigour utilising the Mixed Methods Appraisal Tool and the Authority, Accuracy, Coverage, Objectivity, Date, Significance checklist for appraising grey literature. Data were mapped and analysed onto the six domains of advanced nurse practitioner practice, and the review was reported in line with Preferred Reporting Items for Systematic reviews and Meta-Analyses guidelines.

**Results:**

All papers included in this review spanned across the last 20 years. In total, 45 papers met the inclusion criteria: quantitative (*n* = 11), qualitative (*n* = 15), mixed methods (*n* = 4), and discussion/clinical cases (*n* = 15) papers. Advanced nurse practitioners in Ireland contribute substantial impacts on management and team competence, clinical-decision making, leadership and professional scholarship, professional values and conduct, communication and interpersonal competence, and knowledge and cognitive competence domains. Advanced nurse practitioners in Ireland enhance healthcare outcomes through expertise, coordination, and patient-centred approaches, emphasising their critical role in healthcare delivery and system improvements.

**Conclusions:**

We have highlighted the active role advanced nurse practitioners play in enhancing patient care, improving management and team coordination, and advancing professional scholarship. These insights have provided a foundation for future research and policy development to optimise the advanced nurse practitioner role.

**What is already known about the topic**

• Advanced nurse practitioners are highly skilled nurses who possess the autonomy to assess, diagnose, plan, and coordinate care, and treat patients across various settings.
• Advanced nurse practitioners are known to improve patient outcomes through their ability to provide high-quality, patient-centered care.
• Advanced nurse practitioners play a crucial role in interdisciplinary teams through communication and coordination of care delivery.
• The role of advanced nurse practitioners has evolved significantly over time, and as the demand for healthcare services grows, the role of advanced nurse practitioners is expected to expand further.

Alt-text: Unlabelled box

**What this paper adds**

• We have offered a holistic understanding of the impact advanced nurse practitioners in Ireland have across various domains of professional practice, including management, clinical-decision making, leadership, professional values, communication, and knowledge.
• We have highlighted the active role advanced nurse practitioners play in enhancing patient care, improving management and team coordination, and advancing professional scholarship.
• We have underscored the importance of advanced nurse practitioners in the Irish healthcare system and provided a foundation for future research and policy development to further optimise their role.

Alt-text: Unlabelled box

## 1 Background

Nursing has undergone significant transformation over recent decades, with advancements in healthcare services, technology, and education reshaping the clinical roles and responsibilities of nurses. In response to these changes and the diverse and evolving healthcare needs of an aging population with more complex health care needs, the scope of nursing practice and clinical roles have expanded significantly. The concept of the nurse as a specialist and advanced practice can be traced to the *American Journal of Nursing*, where an American private duty nurse described specialists in three areas: surgical, paediatric, and obstetrical nursing (De Witt, 1900). The emergence of advanced practice, particularly in roles like advanced nurse practitioners (ANPs), reflect the strategic move to improve healthcare service provision and address both service and patient needs.

While various terms have been used within the literature in reference to advanced practice, generally advanced nurse practitioners are registered nurses who have acquired an expert knowledge base, complex decision-making skills, and clinical competencies for expanded practice, the characteristics of which are shaped by the context or country in which they are credentialed to practice and a master's degree is recommended for entry level (International Council of Nurses, 2020). Advanced nurse practitioners are in a pivotal role to support healthcare provision by advancing evidence-based practice and promoting quality improvement within the clinical healthcare environment, ultimately improving patient outcomes (Casey and O'Connor, 2022). Advanced nurse practitioners have a multi-faceted role that requires the application of relevant leadership, knowledge, and skills to provide high-quality patient outcomes and ensure implementation of evidence-based practice. Advanced nurse practitioners are required to meet domains of competence within their role. In Ireland, the Nursing and Midwifery Board (2017) emphasise the importance of advanced nurse practitioners fulfilling competencies of each domain of advanced practice, which include: professional values and conduct, clinical-decision making, knowledge and cognitive competence, communication and interpersonal competence, management and team competence. However, research remains an underdeveloped aspect of the advanced nurse practitioner's role globally (Harbman et al., 2017). Failing to perform in each domain can hinder the success of advanced practice (Lee, 2021). Therefore, it is essential to embrace and prioritise each domain to add value to the role and the healthcare area. Global bodies and leading authorities advocate that nurses should be proactive in identifying research evidence in terms of appraisal and applying evidence to drive improvements in quality and safe care (World Health Organization, 2020). Inherent in this is the need for continuing professional development for advanced nurse practitioners, which is essential for career progression and maintaining person-centred, safe effective care in the workplace (Harbman et al., 2017). In Ireland, funding for education for candidate advanced nurse practitioners (nurses acting in role, who on completion of the requirements, will be appointed to the post) is available, and this kickstarts their continuing professional development. Advanced nurse practitioners prioritise patients in all activities and effect practice and policy changes whenever necessary and hold a unique position, bridging the gap between medical and nursing care, transitioning from expert clinicians to influential leaders in multidisciplinary teams (Thompson and Mcnamara, 2023).

A range of challenges has been identified for low levels of research by advanced nurse practitioners, and these include lack of protected time to engage in research, lack of confidence and competence in research skills and application, and lack of support and resources (Casey and O'Connor, 2022; Strachan et al., 2022). Recognition of the importance of research, education, and leadership in advanced nurse practitioners’ roles is essential for success within their role and their impact within healthcare practice. Journal and research clubs are widely recognised for improving research skills and could be a proposed solution to overcome research barriers by enhancing knowledge, confidence, and skills among advanced nurse practitioners. Collaborative initiatives between academia and clinical settings, such as journal and research clubs, play a pivotal role in translating research into practice, thereby driving evidence-based care and fostering service improvement. These clubs address challenges faced by advanced nurse practitioners regarding research participation by enhancing their knowledge, confidence, and research skills (McConkey et al., 2023). Additionally, they bridge the gap between research and practice by facilitating the application of new knowledge to bedside care, ultimately improving patient outcomes and advancing evidence-based practices. This underscores the effectiveness of journal and research clubs in supporting advanced nurse practitioners-led service innovation and improvement. One such initiative involved a journal and research club comprising eight advanced nurse practitioners, guided by three academic facilitators. The journal club set a primary goal of developing participants' research and appraisal skills. To achieve this, and to better understand the current evidence on the contribution of advanced nurse practitioners to nursing practice and patient care in Ireland, the club undertook the objective of conducting an integrative review. We aimed to conduct an integrative review of the contribution of Irish advanced nurse practitioners to healthcare provision in the 21st century, focusing on the core advanced nurse practitioner domains and impact on nursing practice and patient care.

## 2 Methods

### 2.1 Aim

To identify the contribution of advanced nurse practitioners to care provision in Ireland.

### 2.2 Design

**Fig. 1:**
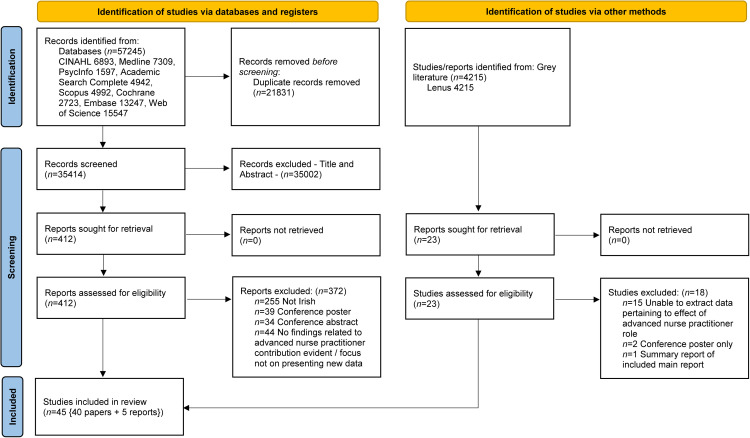
PRISMA 2020 flow diagram.

An integrative review methodology was chosen to identify the current evidence on the topic. Integrative reviews allow for the synthesis of data from various research methods, enabling the generation of new perspectives and a comprehensive understanding of the phenomenon. We followed the systematic approach outlined by Whittemore and Knafl (2005), which is commonly used in nursing. This incorporated five stages and Doody et al's. (2017) 14-stage framework for synthesising qualitative and quantitative data. The PRISMA checklist (Supplementary Material File 1) and PRISMA flow diagram (Fig. 1) were utilised to report this review (Page et al., 2021). The review was registered on Open Science Framework (Doody et al., 2023).

A systematic search was conducted across eight academic databases relevant to nursing and healthcare (CINAHL, Embase, PsycINFO, Scopus, Medline and Academic Search Complete, Cochrane, Web of Science) and database search outputs are available on figshare (Doody et al., 2024). Searches were constructed using three keyword search strings, and Boolean operators (OR/AND) were employed to broaden and combine search strings (Table 1) The search was conducted on the 11th of September 2023. Additionally, hand searching of reference lists and citations of relevant papers (backward and forward chaining), and a grey literature search was performed in Lenus: the Irish Health Repository to ensure inclusivity and mitigate potential indexing issues and inconsistent search terminology (Whittemore and Knafl, 2005).

**Table 1:** Search.

S1	TI (nurse specialist OR advanced practitioner OR advanced nurse practitioner) OR AB (nurse specialist OR advanced practitioner OR advanced nurse practitioner)
S2	TI (assessment OR intervention OR evaluation OR experience OR outcome OR care) OR AB (assessment OR intervention OR evaluation OR experience OR outcome OR care)
S3	S1 AND S2
S4	Limiter from 01 -01–2000

### 2.3 Search outcomes

The results from the database searches were exported to Rayyan (Qatar Foundation, 2022), to facilitate screening for relevance. The search yielded 57,245 results and 21,831 duplicates were identified. The remaining 35,414 were then screened at the title and abstract stage, with 35,002 excluded, leaving 412 for full-text review. The grey literature search yielded 4215 results, and title and abstract screening identified 23 for full-text review. Forward and backward reference chaining did not identify any additional results; 40 papers were included from the database and five from the grey literature. All results were doubled-screened and adjudicated by a third reviewer if a conflict arose. The 45 papers that resulted from the selection process and the reasons others were excluded are presented in Fig. 1. Throughout the process, screening was conducted by paired reviewers to determine eligibility for inclusion, and papers were included if they addressed the criteria of: relating to nursing or midwifery, identifying an advance nurse or midwife practitioner, papers specific to Ireland, and published in English.

### 2.4 Data appraisal

The methodological details of each paper were extracted into a data extraction table (Table 2), and each paper was assessed for quality and rigour utilising the Mixed Methods Appraisal Tool (Hong et al., 2018)) and the Authority, Accuracy, Coverage, Objectivity, Date, Significance checklist for appraising grey literature (Tyndall, 2010). The Mixed Methods Appraisal Tool facilitates a variety of research approaches to be appraised utilising one tool, and the grey literature was assessed utilising Authority, Accuracy, Coverage, Objectivity, Date, Significance, which considers authority, accuracy, coverage, objectivity, date, and significance. Each paper and report was scored as a ‘Yes’, ‘No’ or ‘Can't tell (Supplementary Material File 2). All papers were appraised by paired reviewers to form a consensus, and all papers scored highly.

**Table 2:** Data extraction table.

Author/s, Year	Focus	Design (methods and methodology)	Contribution to practice	Key message/recommendations
Baby, (2019)	Focus on role and practice of advanced midwifery practice in Ireland.	Presents a discussion on advanced midwifery practice and presents data on advanced midwife practitioners practice from the national maternity hospital.	Undertake and document a complete episode of care, perform and read ultrasound, order or perform blood tests, medication prescribing, offering advice and self-help interventions. Staff training and mentoring, collaboration with healthcare professionals. Conduct audits and evaluate service. Ensure safe, evidence based, timely equitable care.	Advanced midwife practitioners provide direct and indirect care.
Begley et al. (2010)	To evaluate advanced nurse practitioner and clinical nurse specialist roles in Ireland.	A mixed methods study consisting of three-phases involving a literature review, focus groups, Delphi study, survey, observations, documentary analysis, and case study with stakeholders’ form post and non-post holder services. Ethical approval obtained.	Care of service users through physical and psychosocial interventions, with early diagnosis and holistic assessment and appropriate referral. Improved patient outcomes. Education. Health promotion. Emotional support and personalised care, resulting in improved therapeutic relationships. Reduced readmission rates, increased adherence to best-practice guidelines, reduced complications, increased continuity of care, increased patient access to care, increased patient satisfaction, teaching/counselling/listening, coordination of care, community resource access and holistic care. Involved in policy development, strategic planning, and service expansion and development. Leadership. Implementing research’ and ‘promotes evidence-based practice. Conferences and publications.	The majority of the advanced nurse practitioners have complex roles and work closely with a multidisciplinary team. The evidence shows positive effects on care quality and costs, and recommends expanding advanced nurse practitioner roles, especially in chronic disease management and community care.
Begley et al. (2013)	To compare the roles, responsibilities, and perceived outcomes of clinical nurse specialists, clinical midwife specialists, and advanced nurse practitioners in Ireland.	Mixed methods study. 6 advanced nurse practitioners, 17 Clinical Nurse Specialist/Clinical Midwife Specialist. Non-participant observation and a three-round Delphi study. Qualitative analysis and descriptive and inferential statistical analysis. Ethical approval obtained.	The advanced nurse practitioners had greater autonomy to make clinical decisions and could therefore process patients through the system more efficiently. Advanced nurse practitioners referred patients/clients to other healthcare workers as appropriate and took referrals from other clinicians. Shorter waiting times experienced and advanced nurse practitioner has greater decision-making skills. advanced nurse practitioners demonstrate clinical leadership by being positive role models in autonomous clinical decision-making. Advanced nurse practitioners practised at a higher level and gave more holistic care. Advanced nurse practitioners were active in teaching and developing educational modules nationally, contributed to national and international guideline development, and sat on high-level national and international committees. Advanced nurse practitioners undertook research, published their work, gave presentations, and attended conferences.	Advanced nurse practitioner roles provide several strategic advantages such as improved service delivery, greater clinical and professional leadership, increased research, and a clear governance and accreditation structure. Development of advanced nurse practitioner posts should be a key focus of the Irish Health Service Executive for the future, in line with its transformation plans for increased community care and support of long-term illness.
Begley et al. (2014)	To explore the views held by key healthcare policymakers on the impact of clinical specialist and advanced practice nursing and midwifery roles.	A qualitative, descriptive design utilising interview of 12 policymakers. Data analysis using constant comparative method. Ethical approval obtained.	Provided an added value and continuity of care. Support clinical practice, particularly psychosocial and practical skills, and improve patient/client outcomes. Provide a swifter access to services, which allowed service-users to be assessed, diagnosed, treated and discharged, thus decreasing waiting lists and waiting times. Organisational skills and follow-up, leading to improved care and compliance. Evidence-based clinical knowledge and a better safety focus. Demonstrate strong clinical leadership, leading in aspects such as guideline development and new care initiatives:	Research output not always visible and protected time, and a stronger link between practice and academia needed.
Begley et al. (2015)	To explore whether clinical specialists in Ireland were fulfilling role expectations in terms of their involvement in audit, evidence-based practice, and research activities.	A case-study design, using survey, observation, interview, documentary analysis, and field notes. 23 matched sites (clinical nurse specialist/ advanced nurse practitioners - non- clinical nurse specialist/ advanced nurse practitioner). Framework analysis. Ethical approval obtained.	Advanced nurse practitioner more effective in implementing evidence-based practice; conducting audit; and leading, conducting, and disseminating research o advance nursing and midwifery practice.	Continued government support for research activity in the health service is necessary if individuals working in the health service are to offer the highest standards of care to service users across a range of services and locations. Have experience as lead researchers, but a lack of time available to publish work.
Behan et al. 2023)	To describe the management of a 39-year-old woman with intractable focal epilepsy during pregnancy who required emergency neurosurgery.	Discussion paper presenting a single clinical case involving an advanced nurse practitioner.	Provided epilepsy and pregnancy education, caring for herself, seizure triggers, sudden, unexpected death of someone with epilepsy and baby care post-partum were provided. Advice on medication management and titration. Coordinated care between centres/services.	There is a need to establish care pathways e.g. between the advanced nurse practitioner-run women's health clinic, the epilepsy surgery service, and the obstetrical epilepsy service.
Blanchfield and McGurk, (2012)	To discuss the establishment of an advanced nurse practitioner renal and diabetes service.	Development of an advanced nurse practitioner service utilising clinical audit, patient satisfaction survey. Analysis and ethics not specified.	Undertake annual review which includes, medical history, education review, and targeted system review including foot examination, fundoscopy, assessment of blood pressure, lipid profile, glycaemic control, renal function and anaemia status. Advanced nurse practitioner diabetes service has a much lower patient non-attendance rate. Deliver continuity of care which is personalised to their individual needs.	Advanced nurse practitioner service has comparable outcomes to standard diabetes care. Consideration should be given by planners and providers to examine new and innovative ways of delivering quality health care meeting the needs of the provider and participant in care.
Blanchfield and O'Connor, (2022)	To profile the development, implementation and evaluation of an alternative combined type 2 diabetes and chronic kidney disease care delivery strategy provided in the context of advanced practice nursing.	Observational design incorporating quantitative (retrospective chart review) and qualitative (focus group interviews) methods. Descriptive statistics and reflexive thematic analysis. Ethical approval received.	Responsibility for the provision of feedback lay with the advanced nurse practitioner. Consultants working with advanced nurse practitioner services identified their potential to contribute to healthcare delivery issues such as lack of access to high-quality personalised care. Advanced nurse practitioners’ ability to initiate treatments contributes to timely management of presenting issues and provision of complete episodes of care. The advanced nurse practitioner contribution was the elucidation of the features of care valued by patient's value and acting as an advocate to incorporate these features in the development and implementation of the combined care service. Advanced nurse practitioners contributed to improved clinical target attainment anaemia management and attenuation of renal function decline.	Services considering combining care provided in the context of advanced practice nursing require resources, expertise with representation from clinical, management, allied health, administrative support and patient stakeholders. If nurses are to contribute to healthcare policy, then a concerted effort is required to demonstrate care and economic outcomes.
Callender and Koe, (2015)	Guidelines developed and advanced nurse practitioner followed guidelines to manage torus fractures with immobilisation in a softcast without orthopaedic referral.	A retrospective chart review of 119 patients and cost analysis. Ethical approval not specified.	There were no adverse events recorded or required subsequent visits to the fracture clinic. No clinically significant complications arose. Cost saving.	Cost saving can be made by review care processes and enabling advanced nurse practitioners to lead care.
Canty and Kearney, (2018)	To explore a quality initiative to improve do not wait for treatment rates implemented by a group of advanced nurse practitioners in an emergency department.	A review of literature and discussion of the initiative rationale, implementation, barriers, and an audit of its effectiveness.	Do not wait for treatment rates were 2.3 % for advanced nurse practitioner-suitable patients, showing a significant reduction (target below 5 %). Patients experience time for advanced nurse practitioner, suitable patients reduced from 2.57 h to 2.37 h (max acceptable 6 h).	Advanced nurse practitioner care provision improves patient experience time and do not wait for treatment rates of patients who are suitable for treatment from advanced nurse practitioners.
Carey et al. (2022)	To identify the ability of an advanced nurse practitioner to practise at a high level of expertise, as autonomous and reach an accurate diagnosis.	Discussion paper including a case report.	Conducting a comprehensive assessment and full review of symptoms using an evidenced based clinical decision-making process. Making a diagnosis. Support advanced care planning. Risk assessment. Pain assessment, medication review, dementia assessment, hydration and constipation assessment. Care management and team collaboration.	Evidence-based algorithms guide clinical practice and facilitate the transfer of research to practice, providing nurses with a step-by-step approach for effective decision-making.
Carthy-Finneran, (2019)	To discuss the advanced nurse practitioner role and how a change in role and focus improves skin cancer patients care.	Discussion paper presenting a single case of an advanced nurse practitioner.	Comprehensive history taking, skin assessment, dermoscopy, clinical examination, dermoscopic evaluation, treatments, would closure suturing, punch biopsy, health promotion, health education, histology management, discharge, surveillance.	There is a need to develop new advanced nurse practitioners and begin succession planning.
Casey et al. (2017)	To explore the perceptions of key stakeholders of the roles of specialist and advanced nursing and midwifery practitioners.	A qualitative phenomenological study of 15 stakeholder (4 advanced nurse practitioners, 3 clinical nurse specialists, 5 policy/management, 3 allied healthcare). Data collected via interviews and analysed using constant comparative technique and thematic analysis. Ethical approval obtained.	Role valued and potential cost saving. Provide a supportive role to colleagues. Positive impact on patient outcomes with greater consistency in care.	Clear and structured career pathway is needed and that individual career pathways must take client and patient needs. Enablers and barriers to these roles related to wide range of issues not lest role ambiguity but also to the lack of, time, collegial and organizational support and workload.
Comiskey et al. (2014)	To explore key patient outcomes and to compare these outcomes across services that employed clinical specialists or advanced nurse practitioners with matched, non-post-holding services.	A national cross-sectional study with a comparison group. 46 services, 23 post-holding and 23 non post holding. Six advanced nurse practitioners, 14 clinical nurse specialists and 3 clinical midwife specialists. Survey. Descriptive and inferential statistical analysis. Ethics approval obtained.	76 % of those attending an advanced nurse practitioner service reported, clinician discussing the service users’ anxieties or fears about their condition. A greater proportions attending advanced nurse practitioners stated that they did not have any anxiety or fears. 98% of service users attending advanced nurse practitioner services had confidence in the clinician. 33% noticing a difference in care received amongst advanced nurse practitioner services. Higher rates reported for positive difference to my health and well-being for those attending advanced nurse practitioner service. The observed mean waiting time was lowest in advanced nurse practitioner sites.	Advanced nurse practitioner roles meet the needs of service users and health service providers should create more of these roles. With increasing emphasis on cost efficiency and faster throughput of users, there is greater need for the reduced waiting times and more holistic care that advanced nurse practitioners can provide.
Coyne et al. (2016)	To explore clinical practice in sites with and without clinical nurse or midwife specialists or advanced nurse practitioners in Ireland.	A case study design. Data collected by interview, observational and documentary data from post holding sites (*n=* 23) and compared with non-post holding sites (*n=* 23). Interviews were held with Directors of Nursing and Midwifery (*n=* 23), healthcare professionals (*n=* 41), service users (*n=* 41) with experience of receiving care or working with postholders, and non-postholders in matched services. The data were analysed using Nvivo. Ethical approval obtained.	Advanced nurse practitioner refer clients to other health care professionals or receive referrals. Reduce re-admission rates by providing advice about symptoms, linking with general practitioners, and identifying when clients needed admission before they deteriorated. Improved continuity of care as permanently in their role. Developing therapeutic communication and good relationships with clients and their carers, by giving time, listened to concerns and showing empathy. Health promotional activities included providing educational materials and practical teaching activities that focused on increasing the client's knowledge. Physical and psychosocial interventions to improve care for clients, such as: symptom management, physical comfort, pain relief, medication and nurse-led clinics, and clients reported a good relationship which led to feelings of trust in their capabilities and trust that they would be seen as a person. Improved health knowledge of clients and carers as they explain the procedures and investigations in clear, understandable language. Promote improved health care by supporting enhanced compliance with treatments, reduced readmissions, prompt treatment and reduction in problems worsening.	Advanced nurse practitioners play a key role in promoting interprofessional team working, which is essential in strengthening healthcare workforce. Advanced nurse practitioner roles should continue to develop in response to changing healthcare needs rather than as replacements for doctors working shorter hours, which have occurred in the past. It is essential that advanced nurse practitioner roles are supported and allowed to expand so that nursing and midwifery workforces are responsive to changing healthcare needs, demographic change, advances in care and treatment, new knowledge and technology, and increased expectations from clients and families.
Elliott, (2010)	To explore how nurses working at advanced practice level in chronic and acute care outpatients responded to decision-making in clinical practice.	A grounded theory study of 21 nurses working at advance practice level. Interviews and constant comparative analysis conducted. Ethical approval obtained.	Therapeutic relationship enabled strategies to be used to engage patients in decision-making and provide information. Relationship assisted in increasing the likelihood that patients would follow the healthcare advice. Monitoring a patient's verbal and non-verbal behaviour. Making a diagnosis. Knowledge of the healthcare system in terms of knowing what treatments were available and how to access them.	The challenge for practitioners is to moderate treatments and present them in such a way that they are accepted by patients.
Elliott et al. (2013)	To report how leadership is enacted by advanced practitioners in nursing and midwifery.	Case study methodology of 23 clinical specialist/ advanced nurse practitioners. Data collected non-participant observation, interviews, and the collection of on-site written records and analysed using an analytic framework. Ethical approval obtained.	Have clinical expertise and central in coordinating patient care and communication with the multidisciplinary team, patient, family, and primary care team Are active in practice development, initiate reviews of clinical practice and patient experiences, identifying specific problems and making changes to patient/client care and service delivery. Responded to new international practice developments and ensured that recommendations from latest research evidence and clinical guidelines are implemented and changes introduced to clinical practice. Influence policy, identify areas for quality improvement initiatives, introduce evidence-based assessment tools and act as consultants to other healthcare organisations. Nurse or midwife-led clinics for specific patient groups including health screening across the lifespan, reviewing patient pathways of care and care processes to improve efficiency of referral and follow-up processes and reduce safety risks. Mentoring, educating and training other staff and undergraduate / postgraduate level.	Advanced nurse practitioner provided valuable leadership across patient care, service provision, policy development, education delivery and team working.
Elliott et al. (2014)	To report a secondary analysis of data collected from the case study phase of a national study of advanced nurse practitioners and develop leadership outcome indicators.	Secondary analysis. All original 23 case study data collect was analysed. Ethical approval for the original study.	Led on some educational interventions. Proactive in the promotion of nursing and has been involved with the curriculum design on several courses. Formal assessment for clinical skills. Knowledge transfer through presentation at research conferences or public patient education media events. Use of evidence and research in clinical practice. Led initiatives for the introduction of new patient services, clinical practices, healthcare processes and support measures that lead to improved patient services and support networks for healthcare practitioners.	Advanced nurse practitioner instrumental in the development patient services and clinical practices. Further validation of the advanced practitioner leadership role components can help to more clearly elucidate the impact they are making.
Gibbons, (2016)	To present a case study of an advanced nurse practitioner dealing with a torn shoulder.	Clinical case study of a 60-year-old man who presented to the emergency department having sustained an injury to his right arm an hour earlier presented.	Physical assessment conducted. Structured and systematic approach to history taking.	Advanced nurse practitioners are increasingly extending and expanding their scope of practice.
Griffin and McDevitt, (2016)	To explore patients’ satisfaction and evaluate the quality of care provided by an advanced nurse practitioner service in an emergence department.	A prospective survey design utilising an adapted questionnaire. 114 participants. Data statistical analysed (descriptives) and open-ended question analysed using content analysis.	100 % agreed that the advanced nurse practitioner seemed to be very thorough. 73.7 % were less worried about their injury after seeing the advanced nurse practitioner. 100 % agreed that they would follow the advice given to them by the advanced nurse practitioner because they believed it to be good advice. 95.5 % agreed that they had enough time to discuss things with the advanced nurse practitioner. 99.1 % would be happy to see an advanced nurse practitioner about a similar injury. 84.0 % were informed of who to contact if they needed more help or advice regarding their injury. 57.1 % were given verbal advice about their injury. 26.8 % were given both verbal and written advice. 86.2 % indicated that the advanced nurse practitioner did enough to help control their pain. 91.9 % thought that the overall quality of care provided by the advanced nurse practitioner service was excellent.	Enhanced history taking and communication skills, are core elements of any advanced nurse practitioner role. Advanced nurse practitioners are providing quality care in extremely busy environments.
Griffin and Melby, (2006)	To determine the attitudes of emergency nurses, emergency doctors and general practitioners towards the development of an advanced nurse practitioner service in Ireland.	A survey design. 21 nurses, 59 doctors. Attitudes Towards Advanced Nurse Practitioner Questionnaire developed. Descriptive and inferential statistical analyses. Ethical approval obtained.	Ninety-four per cent also agreed or strongly agreed that an advanced nurse practitioner service would improve waiting times, and 80 % indicated that it would improve the quality of the existing service. 84 % general practitioners indicated that they would be happy to refer patients with minor injuries to an advanced nurse practitioner and all doctors in the emergency department either agreed or strongly agreed that they would be happy to accept a review from an advanced nurse practitioner. Advanced nurse practitioner service would result in reduced waiting time, and that continuity of care would be enhanced. Both nurses and doctors also thought that this would result in a more cost-effective service.	To enable successful introduction of an advanced nurse practitioner service, the role must be clearly defined, role boundaries must be clarified, and a standardised education, accreditation and monitoring process must be in place.
Health Service Executive, (2016)	To eliminate referrals to the medical teams based advanced nurse practitioner cardiology service.	Discussion based paper utilising example from practice.	Clinical care. Evidence-based interventions. Improving patient flow. Advance physical assessment. Referral system.	Advanced nursing practice fosters creativity and innovation in the delivery of patient-centred care.
Higgins et al. (2014)	To report factors that influence clinical specialists’ and advanced nurse practitioners’ ability to enact their clinical and professional leadership roles.	A case study design. 23 clinical specialists (*n=* 17) and advanced nurse practitioners (*n=* 6), 21 multidisciplinary team, and 13 Directors of Nursing or Midwifery. Interview transcripts, observation and documentary evidence analysed using a qualitative data analysis software NVivo. Ethical approval obtained.	Guides and coordinates the activities of the multidisciplinary team. Initiates and changes patient/client care through practice development. Takes responsibility for policy and guideline development and implementation. Introduces and develops patient/client care services. Changes clinical practice through formal education of the team. Mentors and coaches, the team in clinical practice. Acts as a positive role model for autonomous clinical decision-making and on-going professional development. Develops policy at national and international level. Engages in education outside the service at national and international level. Engages with professional organisations at national and international level.	Building leadership capabilities needs to be embedded in future education plans and sustained through mentorship programmes that account for both clinical and professional leadership at advanced nurse practitioner level. Advanced nurse practitioners are ideally positioned within the clinical team and organisation to lead on the change agenda for health care reform and influence policy at local and national level.
Hunter et al. (2023)	To provide insight into the experiences of women accessing a continuity of care service in Ireland.	A qualitative descriptive study of 11 women. Data collected via interviews and thematically analysed. Ethical approval obtained.	Women experienced this model as a new and welcome approach to maternity care and focused on issues around continuity of care, choice and been involved in care planning.	Not possible for a sole practitioner to maintain the service.
Ingram and Offiah, (2023)	To compare comparing advanced nurse practitioner virtual chest pain clinic to the face-to-face nurse specialist-led clinic.	Retrospective cohort analysis comparing advanced nurse practitioner virtual chest pain clinic (*n=* 102) to the face-to-face nurse specialist-led clinic (*n=* 131).	Patients were assessed, diagnosed, and treated while never actually meeting the advanced nurse practitioner. Advanced nurse practitioner is skilled in obtaining a health history which is the cornerstone in evaluation and diagnosis of patients with chest pain. Advanced nurse practitioner referred significantly fewer patients for further diagnostic tests, showing advanced decision-making skills.	Empowered by advanced nurse practitioner autonomy, a virtual clinic can provide an alternative care pathway.
Ingram et al. (2020)	To describe the post- percutaneous coronary intervention and provide an overview of patient profile and outcomes, with an emphasis on advanced nursing roles and patient symptoms.	This service analysis used patient data to assess and evaluate the nurse-led post- percutaneous coronary intervention clinic. 469 patient appointments. Descriptive and inferential statistical analysis. Ethical approval granted.	Conduct advanced physical assessment and interpret findings. Medication education and management and medication adjustment. Patient education, referral for further investigations or to another specialty. Intervention led by the nurse and discharge into the community.	A comprehensive cardiovascular system examination is an integral part of advanced nursing practice, while the ability to perform a skilled physical examination of the cardiovascular system is essential in providing a nurse-led service.
Ingram, (2017)	A reflection on advanced nurse practitioners’ history-taking skills.	Discussion of health history simulation and a reflection on advanced nurse practitioners’ history-taking skills.	Comprehensive health history, history-taking and enhancing clinical practice.	A commitment to lifelong learning, including self-reflection of personal clinical practice, is a core nursing value.
Leahy and Counihan, (2018)	To establish the extent of concordance in decision-making between the senior attending neurologist and the advanced nurse practitioner in neurology.	A service evaluation. 15 patients triaged by advanced nurse practitioner. Separate pro form as were completed by the advanced nurse practitioner and neurologist and compared. As the study was a service evaluation ethical approval was not needed.	Telephone triage concordance 100 % between the advanced nurse practitioner and the neurologist. Diagnostic concordance 100 % between the advanced nurse practitioner and the neurologist. Management concordance 80 %. Treatment escalation 100 % agreement.	Diagnostic and management decision-making by advanced nurse practitioner for patients with potential relapses and/or treatment escalation is on par with that of the neurologist.
Lennon, (2020)	Focus on role and practice of advanced midwifery practice in Ireland.	Presents a discussion on advanced midwifery practice and presents data on one advanced midwife practitioners’ reflection and data from their practice.	Responsible for providing and co-ordinating care of woman from booking to postnatal discharge. Provide continuity of care and avoid fragmented care. Woman centred care and empower the woman and family to make informed decisions. Operate referral system and collaboration with other healthcare professionals leading to timely review. Order investigations and tests to inform clinical decision making and medication prescribing. Support higher vaginal birth and breastfeeding rate deduced caesarean section rate. Evaluation of service provided.	Advanced midwife practitioners to be a resource for normal-risk pregnancies. Advanced midwife practitioners support the implementation of the national maternity strategy.
McBrien, (2018)	To describe Lisfranc injuries and discuss diagnosis and management.	Discussion paper presenting a single case of an advanced nurse practitioner.	Comprehensive history, physical examination, pain assessment, X-ray requests and referral to consultant.	Advanced nurse practitioners must have a high degree of suspicion when assessing patients who have sustained high impact trauma. Early consultation with an orthopaedic specialist is an essential component of patient care.
McBrien, (2019)	To present a case study to analyse critically the management of a patient with this injury and the care provided by an advanced nurse practitioner.	A clinical case. 1 individual case and analyses of the management and care provided by advanced nurse practitioner in terms of diagnostic decisions and management.	Advanced nurse practitioner performed a history taken (social, family, clinical/medical, medications, work, injury/complaint). Physical examination/assessment, range of movement, pain management. Referral to orthopaedic team.	Advanced nurse practitioner is associated with expert knowledge, complex decision-making and making expert judgements. As advanced nurse practitioners continue to expand their knowledge and scope of practice they can enhance and improve care quality and patient outcomes.
McConkey and Dowling, (2020)	To explore the risks of ionising radiation in individuals of childbearing age attending and advanced nurse practitioner in Urology.	Discussion paper presenting a single case of an advanced nurse practitioner.	A comprehensive health history and focused urological assessment is conducted on each patient presenting to the service. Individual risk assessment is carried out, alternatives explored (decision making) in consultation with the patient (shared decision) in line with policies and guidelines (evidence based). Consultation and referral to consultant.	A comprehensive history and assessment will guide the referrer in adhering to the principles of justification and optimisation, and compliance with local policies and current legislation will promote concordance with best practice.
National Council for the Professional Development of Nurses and Midwifery, (2005)	To provide a preliminary evaluation of the role of the advanced nurse practitioner that will guide the development of the role	A mixed methods approach consisting of a review of documentary evidence, interviews with 8 advanced nurse practitioners, 7 nurse managers, 5 members of multi-disciplinary team, and 7 patients.	Providing holistic care. Leadership. Education. Undertake research. Patient care and outcomes. Teamwork.	Advanced nurse practitioner roles contribute to the development of nursing in the related areas of practice through influence on the practice of others and raising the profile of nursing in that specialty.
National Council for the Professional Development of Nurses and Midwifery, (2010)	To present the profiles of advanced nurse practitioners and clinical nurse specialists.	Descriptive reflective. 7 advanced nurse practitioners described	Expert clinical assessment and holistic nursing care. Improve accessibility of services and reassure. Reduce waiting times. Teamwork. Referrals. Clinical examination, surveillance and follow-up. Education, support and advice. Reduce admissions. Pre-admission. Discharge. Holistic approach. Minimise cancellations. History taking, psychosocial assessment, physical examination, and prescribing X-rays, blood tests, and other diagnostic tests.	Advanced Nurse Practitioner / Advanced Midwife Practitioner roles are developed in response to patient/client need and healthcare service requirements at local, national and international levels and are carried out by autonomous, experienced practitioners who are competent, accountable and responsible for their own practice.
National Council for the Professional Development of Nurses and Midwifery, (2008)	To present the profiles of advanced nurse practitioners and clinical nurse specialists.	Descriptive reflective. 8 advanced nurse practitioners described.	Educate students and others. Manage minor injuries. Reduction in waiting time. Consultancy and managing a caseload. Performs routine procedures. Coordination of follow-up care. Screening. Teamwork. Pain management and treatment. Triage. Patient flow. Patient consultation, assessment, formulation of treatment plans, requesting investigations such as lab work and X-rays, interpretation of investigation results, and ongoing monitoring. Evidence-based high-quality interventions.	Advanced nurse practitioner roles have received strong medical, multi-disciplinary and management support which has been essential in their successful development and implementation.
O'Connor et al. (2018a)	To determine the clinical learning experiences of general practitioners who rotated through an academic urban minor injuries unit as part of their training, led by advanced nurse practitioners (emergency).	A qualitative descriptive study. 5 general practitioners were interviewed and analysed using systematic text condensation. Ethical approval obtained.	Advanced nurse practitioners provide support to general practitioners and are hands-on during the general practitioner's clinical placement. Advanced nurse practitioners provide one-to-one engagement. Supervision by the advanced nurse practitioners (emergency) was also an important role in enabling the trainee general practitioners progress in their performance of particular skills. Advanced nurse practitioners (emergency) operated within their scope of practice utilising evidence-based guidelines and there was no hesitancy on their part to collaborate or refer to medical colleagues if required. Their opinion of the role of the advanced nurse practitioner (emergency) in minor injuries was measured as not only very specialised but invaluable in terms of their command of minor injuries which they share with the general practitioners during their clinical placement.	The advanced nurse practitioner role is very specialised and is invaluable in terms of the provision of supervision, support, learning and feedback for trainee general practitioners throughout their clinical rotation. There are reciprocal learning opportunities for healthcare professionals depending on case complexity.
O'Connor et al. (2018b)	To inform and guide the development of a future model of specialist and advanced nursing and midwifery practice.	Qualitative study. specialist nurses and midwives (*n=* 3), advanced nurse and midwife practitioners (*n=* 4) and key stakeholders (*n=* 8) interviewed. And analysis using constant comparative technique. Ethical approval obtained.	Works at an autonomous level, involved in the whole process of seeing the patient, woman, or client. Diagnosing, prescribing and discharge. Holistic approach that essentially brings the diagnosis and caring aspect of healthcare together in patient outcomes. Interdisciplinary caseload management. Decision-making and a supportive role to nursing and medical colleagues. Reduce length of stay and promote early discharge, quality and safety of care. Make referrals and team-based care approaches.	Advanced practitioner services, which could be expanded to the community and primary care sectors. These services relate to access to comprehensive care, patient flow, hospital avoidance and early discharge from the acute care setting to home
O'Keeffe et al. (2020)	To describes the development of an advanced nurse practitioner led falls pathway in an emergency department to improve safe discharge.	A retrospective 12-month chart review. 77 patients over 65 presented with a fall. Data extraction template used based on NICE guidelines. Descriptive and interpretative statistical analysis. Full ethical approval not required.	Falls assessment conducted (falls screening questions). Discharge where appropriate. Referral to other healthcare professionals and the community falls coordinator. Physical assessment.	Advanced nurse practitioners need to utilise gerontological competencies in line with changing population demographics and increasing complex patient caseloads.
O'Toole et al. (2019)	To evaluate patients’ experiences of and satisfaction with advanced nurse practitioner service.	A 2-site cross-sectional study utilising a survey and retrospective chart review of 117 patients. Statistical analysis and ethical approval obtained.	90.5 % expressed preference to be assessed by the advanced nurse practitioner, rather than being admitted to hospital. 96 % happy with explanations they received about requiring further tests. Satisfaction levels with each aspect of the care pathway was very positive. Advanced nurse practitioners were caring, used language and terms participants understood, and gave them sufficient time at appointments. Advanced nurse practitioner spent more time with them; gave more information, explained it clearer, and listened to them. Shorter waiting time and greater reassurance. Discharge planning.	Advanced nurse practitioner services can guide leaders, policy makers, and clinicians in the reform of emergency service provision.
Richmond et al. (2022)	To explore stakeholders' perceptions of a community-based advanced nurse practitioner -led integrated oral anti‐cancer medication care model.	Qualitative study unspecified. 33 participants (medic 7, clinical nurse specialist 2, nurse manager/leader 4, administrator 1, pharmacist 5, advanced nurse practitioners 5, patients 9). Interviews, focus groups and thematic analysis. Ethical approval obtained.	Advanced nurse practitioners holistic, comprehensive, organised. Strong clinical lead who has expert clinical knowledge and skills for the care and management of this cohort of patients. A source of information and support with expert clinical knowledge and skills for patient care. Corresponding with team members.	Advanced nurse practitioner -led clinic is a means of delivering a professional, quality patient experience within an organised and well-structured environment. Care should be advanced nurse practitioner-led within an integrative approach to support access to the wider hospital-based team.
Rodger et al. (2016)	To develop a programme to promote bone health and falls risk prevention education for health care professionals, clients, families and informal carers.	Action research approach: 1 an audit of outpatient service users, 2 development of bone health promotion resource, 3 an audit of inpatient residential unit, 4 development of falls prevention programme, 5 focus group discussion on bone health and falls risk.	Developed a tailored made educational learning resource. Have been adopted for use by many agencies – schools, colleges, day centres and community groups. Reduction in falls by 33 % since the implementation of the programme. Risk identified and greater awareness among staff.	Need to embrace emerging technologies as they provide scope for educating the general population, healthcare staff, patients and residents.
Small, (2010)	To identify the development of the advanced nurse practitioner role in emergency nursing.	Discussion and reflection on the development of the advanced nurse practitioner role in emergency nursing.	Clinical caseload. Education of others. Comprehensive health assessment. Improved waiting times, patient satisfaction, outcomes and referral pathway expanded.	Advanced nurse practitioners need to maintain competence in clinical practice through continuing professional development.
Thompson and McNamara, (2022a)	To explore how advanced nurse practitioners are positioned within current nursing and health system structures in Ireland.	A qualitative discourse analysis study. Sample of advanced nurse practitioners (*n=* 12), nurses (*n=* 2), clinical nurse managers (*n=* 8), director of nursing (*n=* 1), nursing project officer (*n=* 1), medical practitioners (*n=* 3) and allied healthcare professional (*n=* 2). Interviews and focus groups. Discourse analysis using Gee's Tools of Inquiry. Ethical approval obtained.	The advanced nurse practitioner role as it improves the patient journey through the system. Advanced nurse practitioners have led to significant system improvements and improvements in patient outcomes including reduced waiting times, reduced hospitalisation and reduced lengths of stay. Advanced nurse practitioners see the whole person in their total context, understand more than what is immediately apparent and in turn improve the patient journey and outcomes.	Advanced nurse practitioners are seen to be an expensive commodity, and a situated meaning is constructed that if advanced nurse practitioners do not save money, they cannot be a ‘valuable’ member of the healthcare system. Advanced nurse practitioners approach patient care holistically and more intuitively when compared with other healthcare professionals
Thompson and McNamara, (2022b)	To explore how language works to enable and constrain the role of the advanced nurse practitioner in the health system.	A critical discourse analysis. 29 participants (12 advanced nurse practitioners, 12 nurses, 2 allied health, 3 medical practitioners). Interview, focus groups and field notes. Discourse analysis and ethical approval obtained.	They contribute something unique because they specialise in certain areas so then they become expert in that area. Provide holistic care. Help solve medical problems and waiting lists by diagnosing, treating and discharging.	Hierarchical structures need to be challenged, and this is key to maximising advanced nurse practitioners’ potential and strengthening the unique position they hold in the healthcare system.
Thompson and Meskell, (2012)	To provide empirical evidence for the outcomes of care of advanced nurse practitioners within the emergency department.	A retrospective, comparative audit. 964 patient records and descriptive statistical analyses undertaken.	Advanced nurse practitioner had the lowest rate of false negative results at 0.2 % for X-ray interpretation for fracture. Consultant and advanced nurse practitioner had equal waiting times of 51 mins. Advanced nurse practitioners recognise the need for analgesia administration.	There is a need to describe the precise interventions of advanced nurse practitioners to understand the process and outcomes of their practice.

### 2.5 Data extraction

For data extraction, each paper was read and reread, highlighting relevant details for extraction, and a data extraction table provided a framework to focus and structure the examination of the included papers. Data were extracted by two reviewers for 80 % of papers (*n* = 36), and the remaining 20 % were extracted by the lead author. Data extracted included details on the following: author, year, title, design (methods/methodology), contribution to practice and key message/recommendations. This process enabled data to be extracted and summarised easily.

### 2.6 Data synthesis

To identify landscape and contribution of advanced nurse practitioners in supporting healthcare provision in Ireland and present the results, all data were mapped onto the advanced nurse practitioner six domains of professional practice (Professional values and conduct, Clinical-decision making, Knowledge and cognitive competence, Communication and interpersonal competence, Management and team competence, Leadership and professional scholarship) (NMBI, 2017). All data extracted were coded by holding a team meeting to map all codes onto the six domains of advanced nurse practitioners’ professional practice. Within the coding process, it became apparent that data could span across more than one domain, and, to form an agreement, the coding decision process was guided by the original aim and context of the paper from which the data were extracted, rather than taking the coded data in isolation. This process of mapping the code onto its appropriate domain was conducted using a codebook in Microsoft Excel by two authors (Supplementary Material File 3).

## 3 Results

The search of the databases and grey literature generated 45 papers that met the inclusion criteria and the reasons for exclusion of papers are reported in the PRISMA flow diagram (Fig. 1). The figure presents the characteristics of the studies and the results of the data coding, which are described below in accordance with the six domains of advanced nurse practitioner's professional practice. The domains are presented in numerical order to map the evidence and make recommendations. In total, 511 items were coded onto the six domains; the codes within each domain are presented in Supplementary Material File 3, and the elements of each domain and their sources (papers) are acknowledged in Supplementary Material File 3.

### 3.1 Study characteristics

**Fig. 2:**
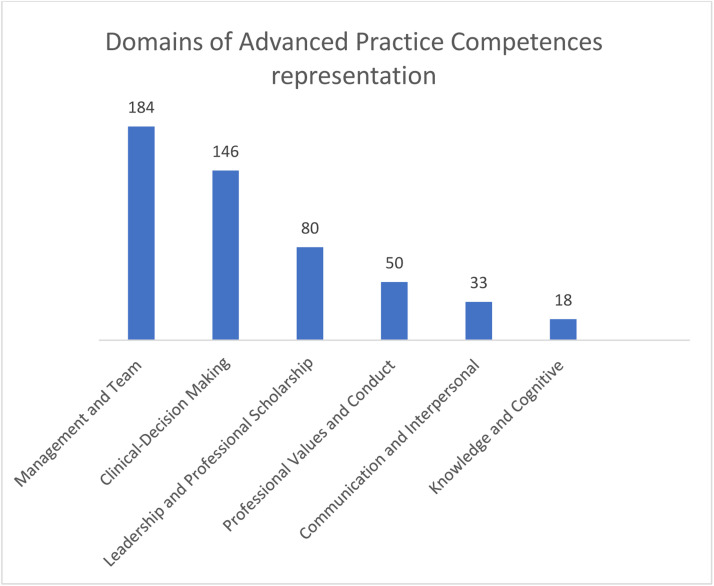
Domain representation.

Papers included in this review spanned in range from 2005 to 2023 (Table 3); 15 papers were non-primary research, 11 quantitative, 15 qualitative, and four mixed methods. Advanced nurse practitioners in Ireland are actively engaging in research and professional development, positively contributing to the individual, family, community, and international body of evidence supporting individualised care provision for persons and their families across the six domains of practice. The management and team domain had the greatest representation (184 codes), with the knowledge and cognitive domain scoring least (18 codes); see Fig. 2. Approximately 17 papers addressed all advanced nurse practitioner practice across Ireland, three were specific to midwifery, two were specific to older persons, and one was specific to outpatients. The remaining 22 papers addressed areas of specialism with nine related to emergency department advanced nurse practitioners, five related to cardiology advanced nurse practitioners, two each related to neurological and endocrine advanced nurse practitioners. Other areas of specialism included endocrine, dermatology, urology, and oncology with one paper each.

**Table 3:** Timeframe and frequency of publications.

Year	Number Published
2005	1
2006	1
2008	1
2010	4
2012	2
2013	2
2014	4
2015	2
2016	5
2017	2
2018	5
2019	4
2020	6
2022	3
2023	3
Total	45

### 3.2 Management and team competence

Management and team were coded 184 times and were evident within 13 elements across 42 studies (Supplementary Material File 3). The most common element was coordination of care and services (27 occurrences); followed by patient journey/experience (23 occurrences); education and training and referral systems (22 occurrences) each; collaboration, team approach, and guidance (21 occurrences) each; waiting time/list (16 occurrences); quality of care (12 occurrences); effective discharge (10 occurrences); health promotion and monitoring and continuity of care (nine occurrences) each; quality care (six occurrences); audit and evaluation (four occurrences); and cost-benefit (three occurrences).

### 3.3 Clinical-decision making

Clinical-decision making was coded 146 times and was evident through five elements across 36 studies (Supplementary Material File 3). The most common element was clinical/client focused care (67 occurrences); followed by assessment (47 occurrences); quality care (14 occurrences); decision-making (10 occurrences); and service provision (eight occurrences).

### 3.4 Leadership and professional scholarship

Leadership and professional scholarship were coded 80 times and were evident through five elements across 20 studies (Supplementary Material File 3). The most common element was leadership, consultancy, and education (27 occurrences); followed by evidence-based and research-based practice (18 occurrences); autonomy of practice (17 occurrences); policy and guidelines development and implementation (10 occurrences); and dissemination of evidence (eight occurrences).

### 3.5 Professional values and conduct

Professional values and conduct were coded 50 times and were evident through 28 elements across 20 studies (Supplementary Material File 3). The most common element was holistic care (nine occurrences); followed by seeing the person, giving of time, therapeutic relationship, and personalised care (three occurrences each); and the elements of listening, involvement in care/care planning, empowerment, caring, patient satisfaction/experience, and providing advice/reassurance, all with two occurrence each. The remaining elements (acting as an advocate, decision-making, emotional support, equitable care, trust, needs based care, individual engagement, professional development, operating within scope of practice, patient care, physical comfort, promoting choice, promoting nursing, providing added value, psychosocial skills, empathy, and being available) all had one occurrence each.

### 3.6 Communication and interpersonal competence

Communication and interpersonal were coded 33 times and were evident through six elements across 12 studies (Supplementary Material File 3). The most common element was providing information and advice (12 occurrences); followed by providing explanation and feedback (seven occurrences); communication across patient, family, and team (five occurrences); alleviating worry and concerns (four occurrences); support and relationship building (three occurrences); and documentation and monitoring (two occurrences).

### 3.7 Knowledge and cognitive competence

Knowledge and cognitive were coded 18 times and were evident through four elements across 10 studies (Supplementary Material File 3). The most common element was expert and specialist knowledge/skills (nine occurrences); while second was supporting knowledge and understanding of patient and carers to enable greater access to services and adherence (four occurrences); and third was supporting understanding through providing advice and education (three occurrences). The least frequent was knowledge of systems and health (two occurrences).

## 4 Discussion

In this review of advanced nurse practitioners in Ireland, we have offered insights into their role and contribution, highlighting critical domains of practice and identified both strengths and areas for development. What we found aligns with the broader international literature on advanced nurse practitioners. The diversity and scope of the studies included, spanning two decades, reflected the evolving and multifaceted role of advanced nurse practitioners, which has been well-documented in international research (Unsworth et al., 2024; Blair, 2018). We have underscored the importance of embracing and prioritising each of the six domains of advanced nurse practitioner practice, as these domains are not only foundational to the role, but also critical for optimising patient outcomes and advancing healthcare systems. The six domains of advanced nurse practitioner practice used in Ireland are consistent with frameworks used in other countries to guide practice and evaluate the contributions of advanced nurse practitioners. For instance, similar domains are emphasised in the United States of America, Canada, the United Kingdom, and Australia (Wheeler et al., 2022). What is evident in this review and the literature is that advanced nurse practitioners are recognised for their critical role in healthcare delivery, particularly in areas like clinical-decision making, leadership, and the management of complex care systems (Lockwood et al., 2022).

The prominent representation of the management and team domain (184 codes) highlights the significance of coordination of care and services in advanced nurse practitioner roles. This mirrors international literature, where advanced nurse practitioners are frequently highlighted for their role in improving care coordination, enhancing patient outcomes, and reducing healthcare costs (Thompson and Mcnamara, 2023; Moxham and McMahon‐Parkes, 2020). The emphasis on coordination, patient journey, education, and referral systems in this domain is particularly relevant, as these elements are critical to the effective functioning of healthcare teams (Moxham and McMahon‐Parkes, 2020). With the highest representation in the data, this domain reflects advanced nurse practitioners’ pivotal role in streamlining care pathways, reducing wait times, and improving care coordination. We have validated the need for sustained investment in resources and interprofessional collaboration to support this aspect of practice.

The substantial focus on clinical-decision making (146 codes) reflects the core function of advanced nurse practitioners in making autonomous clinical judgments, which is a key component of their role internationally (Lockwood et al., 2022). We have highlighted the focus on client-focused care and assessment, which aligns with the literature that positions advanced nurse practitioners as pivotal in providing high-quality, patient-centered care (Carney et al., 2023). The emphasis on client-focused care and assessment highlights advanced nurse practitioners’ autonomy and expertise in delivering patient-centered care. Future priorities include further enhancing decision-making through targeted education and the integration of advanced technologies, such as decision-support systems.

The role of advanced nurse practitioners in leadership and professional scholarship (80 codes) highlights the importance of the advanced nurse practitioner role and is well-supported by international literature (Duignan et al., 2024). Advanced nurse practitioners are often leaders in clinical practice innovation, policy development, and the integration of evidence-based practice into clinical settings. The emphasis on consultancy, education, and research-based practice in this review echoes findings from researchers who recognised advanced nurse practitioners as key drivers of change and innovation within healthcare systems (Fealy et al., 2018). Advanced nurse practitioners are leaders integrating evidence-based practice and driving innovation in healthcare delivery. Building a culture of research and evidence dissemination is essential to maintain this momentum and elevate advanced nurse practitioner practice. Advanced nurse practitioners must continue to engage in clinical and academic leadership roles, advocating for policy changes and leading service improvements.

The focus on professional values and conduct (50 codes) aligns with the growing body of evidence that highlights the ethical and values-driven nature of advanced nurse practitioner practice. The elements of holistic care, patient empowerment, and advocacy are central to advanced nurse practitioner roles across different healthcare systems, reinforcing the importance of maintaining high standards of professional conduct in nursing (Zhang, 2024). The focus on professional values and conduct provides an important lens through which the ethical and compassionate foundation of advanced nurse practitioner roles can be examined. The aspects of holistic care, patient empowerment, and advocacy underscore the ethical nature of advanced nurse practitioner practice. These attributes are indeed pivotal across healthcare systems, as they not only uphold the integrity of nursing but also enhance patient trust and satisfaction. Through connecting these values to high standards of professional conduct, advanced nurse practitioners in Ireland can play a critical role in advancing patient-centered care and supporting ethical decision-making.

The relatively lower coding frequency for communication and interpersonal skills (33 codes) may reflect the challenge of quantifying these inherently qualitative aspects of advanced nurse practitioner practice. However, international literature consistently emphasises the importance of effective communication in nursing, particularly in building trust, supporting patient autonomy, and ensuring the delivery of compassionate care (Eriksson et al., 2021; Kinchen, 2021). While less represented in the findings, effective communication remains fundamental. Advanced nurse practitioners must continue to foster trust and support patient autonomy, emphasising relationship-building and compassionate care.

The least represented domain, knowledge and cognitive skills (18 codes), highlights a potential area for further development and research in advanced nurse practitioner practice in Ireland. While this domain is critical to the role of advanced nurse practitioners, especially in specialised areas, the lower coding may suggest either an underreporting of these skills or a need for more focused research on the cognitive aspects of advanced nurse practitioner roles in Ireland. As the lower represented domain, this suggests a need for ongoing professional development and specialised education in Ireland. Enhancing cognitive skills through targeted initiatives can help Irish advanced nurse practitioners address complex clinical challenges and stay at the forefront of their fields. Internationally, there is strong evidence supporting the importance of continuous professional development and specialised knowledge in enhancing the quality of care provided by advanced nurse practitioners (Casey et al., 2017).

A strength of this review is the insight gained into advanced nurse practitioners’ care provision; however, we recognise that we specifically focused on advanced nurse practitioner roles and may have eliminated articles relevant to the broader areas of advanced practice. Furthermore, we highlighted the dearth of literature specifically relating to advanced nurse practitioners and the views of stakeholders (families, patients). Moreover, we must appreciate that it is challenging to recognise and report on the actions of others, especially those sensitive to the role of nurses, as often their contribution is indirect (e.g., upskilling others) and may be evident only sometime after their intervention. Furthermore, advanced nurse practitioners work as part of a team, thereby making it difficult to attribute changes in outcomes to the advanced nurse practitioner alone.

## 5 Limitations

To the best of our knowledge, this is the first review focusing on advanced nurse practitioners in Ireland. While it provides valuable insights, several limitations should be acknowledged. Firstly, the included papers varied in methodological rigor and quality. Some were based on small sample sizes, self-reported data, or descriptive designs, which may limit the strength of the evidence. Secondly, while this geographical focus was intentional (Ireland only), it may limit the generalisability of the findings to other countries or healthcare systems with differing regulatory, educational, and organisational frameworks for advanced nurse practitioners. Thirdly, papers included diverse settings, specialisations, and methodologies, making it challenging to synthesise findings into a unified narrative. Fourthly, papers were cross-sectional in nature, and one-third-of the papers in the review were discussion/clinical cases, limiting the ability to assess the long-term impact of advanced nurse practitioners on healthcare provision and patient outcomes. Despite these limitations, we have highlighted significant contributions of advanced nurse practitioners to Irish healthcare and provided a foundation for further research and policy development in this area.

## 6 Conclusions

We provided a comprehensive mapping of the roles and contributions of advanced nurse practitioners onto to domains of competence that allow for registration in Ireland as an advanced nurse practitioner; these are consistent with international trends. The emphasis on management, clinical-decision making, and leadership aligns with global research that positions advanced nurse practitioners as critical players in modern healthcare systems. However, we also highlighted areas for further exploration, particularly in the domains of communication and interpersonal skills, and cognitive expertise. Future researchers could further investigate these areas, drawing comparisons with international best practices to continue advancing the role of advanced nurse practitioners in Ireland.

## CRediT authorship contribution statement

**Owen Doody:** Writing – review & editing, Writing – original draft, Validation, Project administration, Methodology, Investigation, Formal analysis, Data curation, Conceptualization. **Margaret Graham:** Writing – review & editing, Writing – original draft, Validation, Methodology, Formal analysis, Data curation, Conceptualization. **Orla Hegarty:** Writing – review & editing, Writing – original draft, Methodology, Formal analysis. **Anne Marie Sloane:** Writing – review & editing, Writing – original draft, Methodology, Formal analysis, Conceptualization. **Pauline Walsh:** Writing – review & editing, Writing – original draft, Methodology, Formal analysis, Conceptualization. **Mary Synnott:** Writing – review & editing, Writing – original draft, Conceptualization. **Mary Russell:** Writing – review & editing, Writing – original draft, Conceptualization. **Trudy Dunworth:** Writing – review & editing, Writing – original draft, Conceptualization. **Jill Sheridan:** Writing – review & editing, Writing – original draft. **Louise Murphy:** Writing – review & editing, Writing – original draft, Methodology, Formal analysis, Data curation, Conceptualization.

## Declaration of competing interest

The authors declare that they have no known competing financial interests or personal relationships that could have appeared to influence the work reported in this paper.

## Appendix Supplementary materials

Image, application 1

Image, application 2

Image, application 3
